# Estimating trematode prevalence in snail hosts using a single-step duplex PCR: how badly does cercarial shedding underestimate infection rates?

**DOI:** 10.1186/1756-3305-7-243

**Published:** 2014-05-27

**Authors:** Ana Born-Torrijos, Robert Poulin, Juan Antonio Raga, Astrid Sibylle Holzer

**Affiliations:** 1 Cavanilles Institute for Biodiversity and Evolutionary Biology, Science Park, University of Valencia, PO Box 22 085, 46071 Valencia, Spain; 2 Department of Zoology, University of Otago, P.O. Box 56, Dunedin 9054, New Zealand; 3 Institute of Parasitology, Biology Centre of the Academy of Sciences of the Czech Republic, Branišovská 31, 370 05 České Budějovice, Czech Republic

**Keywords:** Prevalence, Detection, Snail host, Double infection, Single-step duplex PCR

## Abstract

**Background:**

Trematode communities often consist of different species exploiting the same host population, with two or more trematodes sometimes co-occuring in the same host. A commonly used diagnostic method to detect larval trematode infections in snails has been based on cercarial shedding, though it is often criticized as inaccurate. In the present study we compare infection prevalences determined by cercarial emission with those determined, for the first time, by molecular methods, allowing us to quantify the underestimation of single and double infections based on cercarial emission. We thus developed a duplex PCR for two host-parasite systems, to specifically differentiate between single and double infections. The Ebro samples include two morphologically similar opecoelids, whereas the Otago samples include two morphologically different larval trematodes.

**Methods:**

Snails were screened for infections by incubating them individually to induce cercarial emission, thus determining infection following the “classical” detection method. Snail tissue was then removed and fixed for the duplex PCR. After obtaining ITS rDNA sequences, four species-specific primers were designed for each snail-trematode system, and duplex PCR prevalence was determined for each sample. Results from both methods were statistically compared using the McNemar’s Chi-squared test and Cohen’s Kappa Statistic for agreement between outcomes.

**Results:**

Overall infection prevalences determined by duplex PCR were consistently and substantially higher than those based on cercarial shedding: among Ebro samples, between 17.9% and 60.1% more snails were found infected using the molecular method, whereas in the Otago samples, the difference was between 9.9% and 20.6%. Kappa values generally indicated a fair to substantial agreement between both detection methods, showing a lower agreement for the Ebro samples.

**Conclusions:**

We demonstrate that molecular detection of single and double infections by duplex PCR strongly outcompetes the classical method. Detection failure is most likely due to immature and covert infections, however, the higher incidence of misidentified double infections in the Ebro samples arises from morphological similarity of closely-related species. The higher accuracy of the duplex PCR method also adds to our understanding of community structure of larval trematodes in snail hosts, by providing a clearer assessment of the importance of interspecific interactions within the host.

## Background

Trematode communities often comprise several different species that exploit a single snail host population, with two or more trematode species sometimes co-occurring in the same individual host [1,2]. The prevalence of double or multiple infections is usually very low, suggesting that interspecific competition or some other form of negative interaction greatly limits the co-occurrence of more than one species per individual host [1,3,4]. Other processes can affect the prevalence of double infections [2]. Sewell [5] proposed that parasitized snails lose their chemical attractiveness to other searching parasites, with the original parasite perhaps altering the snail’s physiology to impede or prevent the development of later infections. This might prevent certain combinations of co-occurring species and allow others [6]. Also, infection by a second species could be facilitated due to the suppression of the snail’s resistance mechanism [7]; infection with one species of trematode may then predispose some molluscs to infections with another species [8]. Overall, the generally low frequency of double infections suggests that antagonistic relationships between trematodes play important roles. However, any inference regarding interspecific interactions or the structure of trematode communities in snail hosts depends on accurate methods to detect infections in snails.

One commonly used diagnostic method for the detection of larval trematode infections in snail intermediate hosts has been based on cercarial shedding. Usually, snails are individually isolated in small containers and incubated for some time under constant illumination and temperature. After this, emerged cercariae are identified under the stereomicroscope and their prevalence is recorded. In order to increase the accuracy of this non-destructive method of detection and also detect latent or covert infections with immature parasites, many researchers use either (i) multiple sequential sheddings over a period of days or weeks, thereby allowing cercariae time to mature [9], or (ii) subsequent dissection of snails [9,10]. In studies where live snails are not needed, only dissection may be used [11,12]. Other methods are rarely used as they are time-consuming, for example the enzymatic electrophoresis of snail digestive glands which allows detection and identification of immature infections ([13], see references in [14]).

Not surprisingly, cercarial release as a detection method has been criticised as inaccurate by several authors [9,15-19]. In studies where prevalence estimates obtained from both cercarial release and snail dissection were compared, the prevalence was higher with the latter method [15-17,20], including the detection rate of multiple infections [17,21]. Moreover, in some cases snails containing mature cercariae did not shed any [22].

Furthermore, it was shown by Curtis and Hubbard [17] that screening for cercarial release in snails with mixed infections is a conservative approach that only identifies mature infections and thus underestimates the true prevalence [9,17]. The detection of double infections is also more difficult as the simultaneous production of cercariae by two species in the same snail is lower than what they achieve in single infections [23-25], possibly due to competition for host resources.

To quantify the underestimation of trematode prevalences based on cercarial emission, in the present study we investigated two host-parasite systems with double infections by comparing infection prevalences determined by emission with those determined, for the first time, by molecular methods. Several earlier studies [15-17,20] have already compared the results obtained by cercarial emission with those obtained by dissection, and found significant differences. Our goal was to compare the results obtained by emission with those of a method even more powerful than dissection, to ascertain the ‘true’ number of infections that are missed by relying on cercarial emission alone. Despite their use for differentiation between species, molecular methods have so far only been used for comparison of the ‘true’ infection prevalences with those obtained with the ‘classical’ cercarial shedding method, in single infections [19]. Molecular methods are yet to be applied to the detection of mixed infections, where immature and covert infections may be more common.

Caron *et al.*[14], in a review of the techniques used for investigating infection levels in snails already highlighted the importance of PCR-based techniques. We thus developed a duplex PCR assay for two host-parasite systems, capable of specifically amplifying differentially sized segments of the internal transcribed spacer region of ribosomal DNA (ITS rDNA) of each larval trematode species infecting the same snail host (in the digestive glands or gonads), and differentiating between single and double infections. The two systems include i) two co-occuring trematode species both with sporocysts as their intramolluscan stages, and ii) two co-occurring species, one with rediae and one with sporocysts. The latter combination commonly shows stronger interspecific antagonism [1,7,26,27]. The two host-parasite systems are: 

(i) The Ebro samples: The snail *Gibbula adansonii* (Payraudeau, 1826) (Prosobranchia, Trochidae) occurs in the Western Mediterranean, and acts as first intermediate host of the sympatric species *Cainocreadium labracis* (Dujardin, 1845) (Opecoelidae) and *Macvicaria obovata* (Molin, 1859) (Opecoelidae) [28]. Sporocysts of both *C. labracis* and *M. obovata* infect the snail’s gonad and digestive gland. The prevalence of *C. labracis* in the Ebro Delta varies from 17.6% to 30.8% [28], while for *M. obovata* the prevalence varies from 0.9% to 23.1% [28].

(ii) The Otago samples: The snail *Zeacumantus subcarinatus* (Sowerby, 1855) (Prosobranchia: Batillariidae) is highly abundant in New Zealand in soft-sediment intertidal areas as well as sheltered rocky shores, and acts as first intermediate host of, among others [29], *Philophthalmus* sp. (probably *P. burrili* Howell & Bearup) (Philophthalmidae) and *Maritrema novaezealandensis* Martorelli, Fredensborg, Mouritsen and Poulin, 2004 (Microphallidae) [30]. Rediae of *Philophthalmus* sp. occur in the digestive gland and gonad. Depending on the site of collection, its prevalence varies from less than 5% up to 30% [9]. Sporocysts of *M. novaezealandensis* can be found in the gonad, with a prevalence that varies among sites from less than 5% to over 80% [9,30,31]. The prevalence of double infections is generally very low, i.e. <2% [9].

## Methods

### Study design

For the study of the two parasite-snail systems, (i) *Gibbula adansonii* were collected by hand on seven different occasions at the “Beach of the Eucalyptus” (40°37′35″N, 0°44′31″E) in Els Alfacs lagoon (Ebro Delta, Spain) between March and May 2011, December 2011, and between March and May 2013, and (ii) individuals of *Zeacumantus subcarinatus* were collected by hand on four different occasions in Lower Portobello Bay (45°49′56′′S, 170°40′22′′E) and Oyster Bay (45°50′21′′S, 170°38′33′′E) (Otago Habour, New Zealand) between December 2012 and February 2013.

After two days of acclimatisation to laboratory conditions, the snails were screened for infections by incubating them individually in cell wells containing 3 ml seawater, at 25°C with illumination for (i) 14 h followed by a dark period of 10 h in the case of *G. adansonii*, and for (ii) 3 h in the case of *Z. subcarinatus*, thereafter checking for the presence of emerged cercariae. The illumination times differed because one species of cercariae shed from *G. adansonii* emerges more during dark periods [32], whereas larval trematodes infecting *Z. subcarinatus* emerge within 2-6 h of constant illumination [9,27,33].

Incubation was carried out at 25°C as warmer temperatures promote cercarial emission, a general phenomenon also documented for our study species [32-38]. After the incubation, individual wells were examined under a dissecting microscope for the presence of cercariae, to ascertain infection status. The few snails infected by other trematode species were discarded, while all remaining snails, whether or not they shed cercariae of our focal species, were then also used for the second detection method, the ‘duplex PCR method’. A total of 257 *G. adansonii* and 287 *Z. subcarinatus* were used for the study. The prevalence based on either the ‘classical’ detection method or the duplex PCR method was calculated as the number of infected snails divided by the total number of snails examined. For the duplex PCR, the digestive gland and gonads of all snails were removed and fixed in 100% ethanol.

For the study, a waiver was granted from the University of Otago and the University of Valencia Animal Ethics Committees, since no formal approval or ethic statement is required for research on gastropods under the New Zealand and the Spanish legislation.

### ITS sequences

The ITS rDNA sequences of the trematodes *C. labracis* [Genbank JQ694148] and *M. obovata* [GenBank JQ694145] published in Born-Torrijos *et al*. [28] were used for the design of specific primers for samples obtained from the snail *G. adansonii*.

New ITS rDNA sequences of the trematodes *M. novaezealandensis* and *Philophthalmus* sp. had to be produced, using two individual specimens for each species. DNA extraction consisted of placing ethanol-dried samples into 300 μl of 5% Chelex containing 100 μgmL^-^1 proteinase K, incubating at 60°C overnight, boiling at 90°C for 8 min and centrifuging at 15,000 g for 10 min. Polymerase chain reaction amplifications (PCRs) were performed with a programmable thermal cycler (Mastercycler ep gradient S, Eppendorf) in a final volume of 20 μl containing ~0.5 units of MyTaqRed DNA Polymerase (Bioline) and the related 5x buffer (MyTaq Red Reaction Buffer system, which includes 15 mM MgCl_2_ and 5 mM dNTPs), 0.5 μM of each primer and approximately 100 ng of template DNA. ITS2 rDNA sequences were amplified using primers 3S (forward 5′-GGT ACC GGT GGA TCA CGT GGC TAG TG-3′) (middle of 5.8S rDNA) [39] and ITS2.2 (reverse 5′-CCT GGT TAG TTT CTT TTC CTC CGC-3′) (5′end of 28S rDNA) [40]. The following thermocycling profile was used for amplification of the gene region: denaturation (95°C for 3 min); 35 cycles of amplification (94°C for 50 s, 54°C for 50 s and 72°C for 1 min 20 s); and 4 min final extension at 72°C. Two PCR amplicons per species were gel-excised and purified using Ultra-Sep Gel Extraction Kit (Omega Bio-Tek), cycle-sequenced from both strands using ABI BigDye™Terminator v3.1 Ready Sequencing Kit, alcohol-precipitated, and run on an ABI 3730 sequencer (Applied Biosystems). The PCR primers were used for cycle sequencing, and contiguous sequences were assembled and edited using Bioedit v7.0.5 (©1997–2005) [41]. The sequences were given a GenBank Accession Number [*M. novaezealandensis* KJ540203 and *Philophthalmus* sp. KJ540204].

### Specific primer design and duplex PCR

For the primer design, an alignment of 17 trematode taxa [GenBank: AJ277372.1, AJ241814.1, AJ241802.1, AJ241817.1, AJ241793.1, AJ241808.1, AJ241807.1, AJ241795.1, AJ241816.1, AJ241798.1, AJ241797.1, AJ241796.1, AJ241800.1, AJ241801.1, AJ241799.1, AJ241794.1, AJ241806.1] for the Ebro samples, and an alignment of 22 trematode taxa [GenBank: JN621323.1, GQ463127.1, GQ463124.1, AJ564384.1, AF336234.1, GQ463138.1, GQ463132.1, AJ564383.1, AF067850.1, HQ650132.1, HM584170.1, HM584172.1, HM584175.1, HM584183.1, HM584198.1, HM584181.1, HM584196.1, HM584190.1, HM584180.1, HM584171.1, FJ211246.1, JF784190.1] the Otago samples, were produced with the newly generated sequences. Variable regions of the ITS rDNA were detected from those alignments. Other trematode species different from our focal species infect the snail *Z. subcarinatus* in the Otago samples, but sequences of *Microphallus* sp., *Acanthoparyphium* sp*.* and *Galactosomum* sp. could not be included in the alignment due to the large percentage of sequence divergence leading to non-reliable alignment.

We designed multiple taxon-specific primers by using the Primer3 program [42] and considering physical and structural properties of the oligonucleotides (annealing temperature ≥ 60°C, G + C percentage over 60%, and self-complementarity, primer dimers and hairpins). Forward and reverse primers for each trematode species were designed (see Table 1). In the process, care was taken that amplicons of co-infecting species were differentiable by size, and that they did not align with other species parasitizing the snail host. To select the appropriate annealing temperature for the 4 primers of each duplex PCR, a temperature gradient was used, with an artificial mixed infection (DNA of two species was combined). For all primers listed in Table 1, duplex PCR conditions were as follows: denaturation (94°C for 3 min); 35 cycles of amplification (94°C for 50 s, 66.6°C (for the Ebro samples) or 64°C (for the Otago samples) for 1 min, and 72°C for 1 min); and 4 min final extension at 72°C. PCR products were visualized on a 1.5% agarose gel, stained with ethidium bromide. The band sizes were checked against a GeneRuler 100 bp Plus DNA Ladder (Thermo Scientific). To confirm the identity of the bands, the amplicons of two duplex PCR products with previously known samples, were gel-excised, purified using the NucleoSpin Gel and PCR Clean-up kit (Macherey-Nagel) in accordance with the manufacturer’s instructions and sequenced. Positive controls consisted of target DNA from an artificial mixed infection, while in negative controls nanopure water was used instead of DNA sample. Parasite prevalence was determined for each separate sample (i.e. each sampling year for the Ebro, each bay sampled for Otago). When amplification was negative for both species, PCR was repeated with a 1:50 dilution of the template, thus eliminating false negatives due to inhibition.

**Table 1:** Species-specific ITS rDNA primers

**Species [GenBank Acc.Number]**	**Annealing temperature**	**Primer name**	**Primer sequence 5’-3’**	**PCR product size (nt)**	**Amplified region**
*C. labracis* [JQ694148]	66.6°C	Caino_F	ACGTGCAGCTCATGACACGG	301	ITS1
		Caino_R	TCAGTCAAGCCAGGGGAAGG		
*M. obovata* [JQ694145]		Macv_F	CCCGAGGCACTCAAAGACTG	537	ITS1
		Macv_R	TCAGTCGAGCCCAGGATAGG		
*M. novaezealandensis* [KJ540203]	64°C	Maritr_F	TTGACATTCGGCCGGGGTGC	214	ITS2
		Maritr_R	ACCGGCCTAAAGCGCACAGA		
*Philophthalmus* sp. [KJ540204]		Philsp_F	CGTGAGAGATCACGCGAGG	352	ITS2
		Philsp_R	TGTGCGCCTCACCAAGTGAG		

Species-specific ITS rDNA primers designed in this study for amplification of i) *C. labracis* and *M. obovata*, infecting *G. adansonii* snails from the Ebro Delta (Spain) and ii) *M. novaezealandensis* and *Philophthalmus* sp., infecting *Z. subcarinatus* snails from Otago Habour (New Zealand).

### Statistical analyses

The results from the classical detection method and the duplex PCR detection method were transformed in two dimensional contingency tables (2 × 2). Statistical differences between prevalences obtained by the two methods were evaluated by McNemar’s Chi-squared test for paired proportion (χ2, critical p-value < 0.05) (R, package stats, version 2.15.0) [43]. Additionally, the Cohen’s Kappa Statistic (K, critical p-value < 0.05) for agreement between both techniques was calculated (R, package fmsb, version 0.4.1) [44], using the estimated Kappa to assess the extent of agreement (following [45]). If Kappa is less than 0, the prevalences obtained from the two methods show "No agreement", if 0–0.2, "Slight agreement", if 0.21-0.4, "Fair agreement", if 0.41-0.6, "Moderate agreement", if 0.61-0.8, "Substantial agreement", and if 0.81-1.0, "Almost perfect agreement". The Cohen’s Kappa tests the null hypothesis that the agreement between the two methods is the same as random, with Kappa =0. A higher Kappa shows a higher extent of agreement. Statistical analyses were conducted in the software R (version 3.0.1 [46]).

## Results

### Prevalences comparison: Classical detection method and duplex PCR

**Figure 1:**
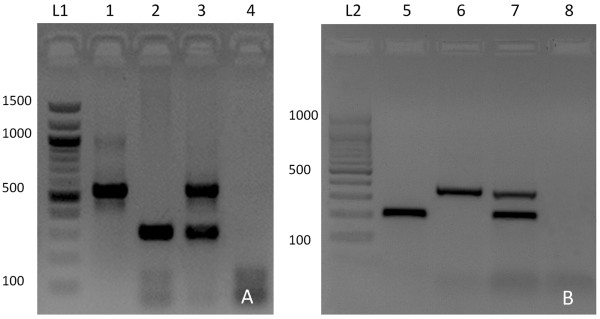
**Single-round duplex PCR detection method for single and double trematode infections in snails.** Agarose gels showing amplicons produced by single-round duplex PCR reactions for single and double trematode infections in snails, based on ITS rDNA sequences: **(A)** PCR products of infected *G. adansonii* tissues (Ebro samples). From left to right, L1 shows a 1500 bp DNA ladder, lane 1 *M. obovata* (537 bp), lane 2 *C. labracis* (301 bp), lane 3 artificially mixed infection, lane 4 negative control. **(B)** PCR products of infected *Z. subcarinatus* tissues (Otago samples). From left to right, L2 shows a 1000 bp DNA ladder, lane 5 *M. novaezealandensis* (214 bp), lane 6 *Philophthalmus* sp. (352 bp), lane 7 artificially mixed infection, lane 8 negative control.

All PCR amplicons, which were sequenced for control confirmed the expected identity of each single and double infection. As shown in Figure 1, infections of the snail tissue samples could be easily differentiated after simple gel visualization.

In both snail-parasite systems, overall infection prevalences determined by duplex PCR were considerably higher than those determined by the classical cercarial shedding method. Depending on the year of sampling (2011 *versus* 2013), among the snails from the Ebro delta 17.9% and 60.1% more snails were found infected using the molecular method. In the Otago samples, the difference between prevalences based on the classical and duplex PCR method was not quite as pronounced but still important with 9.9% and 20.6% more infections detected by PCR, depending on the sampling site (Lower Portobello Bay *versus* Oyster Bay).

Estimates of prevalence of both single and double infections were higher with the duplex PCR method, the difference ranging from 0.6% to 54.2% for single infections and 2.4% to 9.5% for double infections. In the Ebro data (Table 2), the detection rate of single infections with the duplex PCR method was between 1.1% and 3.5% higher for *M. obovata*, and between 7.9% and 54.2% higher for *C. labracis*, the latter species being overlooked in 18.4% and 66.9% of the infections by the classical method. In the Otago data, the detection rate of single infections with the duplex PCR method was between 6.2% and 15.9% higher for *M. novaezealandensis*, and between 0.6% and 2.4% higher for *Philophthalmus* sp., the latter species being overlooked in up to 50% of the infections by the classical method. The increased detection of double infections with the duplex PCR method ranged between 9% and 9.5% in the Ebro data and between 2.4% and 3.2% in the NZ data. Given their low frequency, the most important difference lay in the detection of double infections. Indeed, depending on the sample, from 41.6% to 80% of the double infections detected by duplex PCR were not detected by the classical method (Table 2). Only in single infections of *M. obovata* in 2013 (Ebro samples), was the prevalence detected by duplex PCR lower (3.6%) than that of the classical method (7.1%); however, this was due to the detection of double infections in the same snails thus causing a strong increase in the number of double infections in that sample, from 2.4% (classical method) to 11.9% (duplex PCR method).

**Table 2:** Prevalence of infections detected by the classical detection method and the duplex PCR detection method

		**Total no. snails**	**Infection**	**No. infected Emission**	**Prevalence (%) Emission**	**No. infected duplex PCR**	**Prevalence (%) duplex PCR**	**% infections that failed detection by emission**
**Ebro samples**	**2011**	89	*C. labracis*	31	34.8	38	42.7	18.4
			*M. obovata*	21	23.6	22	24.7	4.5
			Double infection	6	6.7	14	15.7	57.1
			Total 2011 infected	58	65.2	74	83.1	21.6
	**2013**	168	*C. labracis*	45	26.8	136	81	66.9
			*M. obovata*	12	7.1	6	3.6	-1
			Double infection	4	2.4	20	11.9	80
			Total 2013 infected	61	36.3	162	96.4	62.3
**Otago samples**	**LP**	161	*M. novaezealandensis*	81	50.3	91	56.5	11
			*Philophthalmus* sp.	10	6.2	11	6.8	9.1
			Double infection	7	4.3	12	7.5	41.6
			Total LP infected	98	60.9	114	70.8	14.0
	**OB**	126	*M. novaezealandensis*	62	49.2	82	65.1	24.4
			*Philophthalmus* sp.	3	2.4	6	4.8	50
			Double infection	1	0.8	4	3.2	75
			Total OB infected	66	52.4	92	73.0	28.3

Prevalence and numbers of infections detected based on the classical method (emission of parasites) and the duplex PCR method. i) Ebro samples: *C. labracis*, *M. obovata* and double infections in *G. adansonii*, from the Ebro Delta (Spain), years 2011 and 2013; and ii) Otago samples: *M. novaezealandensis*, *Philophthalmus* sp. and double infections in *Z. subcarinatus*, from Otago Habour (New Zealand), sampling sites (LP: Lower Portobello Bay, OB: Oyster Bay). Percentage infections not detected by emission = (no. infected duplex PCR minus no. infected classical method) / no. infected duplex PCR.

The two methods were compared using 2 × 2 contingency tables, for each sample and type of infection (Table 3). The results of McNemar’s test revealed statistically significant differences between the prevalences obtained by the two detection methods. The majority of comparisons (7 out of 12) between the two detection methods show that the detection by duplex PCR is significantly higher. The lack of significance of some comparisons (Table 3) is probably due to the modest sample sizes. Kappa values generally indicated a fair to substantial agreement between the classical method and PCR results (22 to 78% of agreement). Importantly, the Kappa values obtained from the Ebro samples show a lower agreement between both detection methods than those of the Otago samples, with double infections showing generally a much lower agreement between the methods. In samples with low agreement between the methods, the strength of that agreement was non-significant, maybe due to the low prevalences of infections (i.e. double infections).

**Table 3:** Comparison of trematode infections by statistical methods

		**Parasite species**	**Classical method detection**	**Duplex PCR detection**		**McNemar**		**Cohen’s Kappa**	
				**+**	**-**	**X**^**2**^**-test**	**P-value**	**K**	**P-value**
**Ebro samples**	**2011**	*C. labracis*	**+**	30	7	6.76	0.01	0.37	<0.001
			**-**	22	30				
		*M. obovata*	**+**	24	4	3.06	0.08	0.61	<0.001
			**-**	12	49				
		Double infection	**+**	3	5	18.27	<0.001	-0.01	0.53
			**-**	32	49				
	**2013**	*C. labracis*	**+**	49	0	63.02	<0.001	0.33	<0.001
			**-**	65	54				
		*M. obovata*	**+**	11	5	2.72	0.1	0.49	<0.001
			**-**	13	139				
		Double infection	**+**	3	1	12.5	<0.001	0.22	0.13
			**-**	17	147				
**Otago samples**	**LP**	*M. novaezealandensis*	**+**	87	1	11.53	<0.001	0.78	<0.001
			**-**	16	57				
		*Philophthalmus* sp.	**+**	16	1	3.13	0.08	0.77	<0.001
			**-**	7	137				
		Double infection	**+**	6	1	2.29	0.13	0.61	0.003
			**-**	6	148				
	**OB**	*M. novaezealandensis*	**+**	62	1	19.36	<0.001	0.6	<0.001
			**-**	24	39				
		*Philophthalmus* sp.	**+**	4	0	4.17	0.04	0.55	0.02
			**-**	6	116				
		Double infection	**+**	0	1	0.8	0.37	-0.01	0.51
			**-**	4	121				

Comparison of trematode infections in snails detected by the classical method (emission of parasites) and the duplex PCR method. i) Ebro samples: *C. labracis*, *M. obovata* and double infections in *G. adansonii*, from the Ebro Delta (Spain), years 2011 and 2013; and ii) Otago samples: *M. novaezealandensis*, *Philophthalmus* sp. and double infections in *Z. subcarinatus*, from Otago Habour (New Zealand), sampling sites (LP: Lower Portobello Bay, OB: Oyster Bay). Data transformed in two-dimensional contingency tables (2×2). Results from McNemar’s Chi-squared test for paired proportions (χ2) and Cohen’s Kappa Statistic (K) for agreement between both methods. Kappa values can range from <0 (no agreement) to 1 (perfect agreement).

### Unusual events

Fourteen infections detected by the classical method had an unusual duplex PCR outcome in the Ebro samples: 1. Cercarial emission was identified as *C. labracis* but duplex PCR detected only *M. obovata* (four samples, 1.6%), 2. The opposite case (seven samples, 2.7%), and 3. Double infections detected by emission were identified as single *M. obovata* infections by duplex PCR (three samples, 1.2%). In the Otago samples, only one unusual event occurred: Two snails identified with double infections by cercarial emission were determined to be single infections by duplex PCR (one each for *M. novaezealandensis* and *Philophthalmus* sp. respectively, 0.7%).

## Discussion

Traditionally, studies evaluating the influence of parasitism on snails or changes induced in parasitized snails determine the infection status of snails used in experiments based on parasite emission following incubation (i.e. the classical method). Additionally snail dissection may be used, and provides more reliable results than those based on cercarial emission [15-17,20]. However, as highlighted here, these methods may not be accurate enough, especially for the detection of immature and double infections. More recently, some studies have used species-specific primers for the identification of single larval individuals [47], and many studies use molecular methods for parasite detection and identification (as example [48-50]), quantification of infection levels within a host [51], or co-infection prevalence of trematode eggs in stool samples [52,53]. Martínez-Ibeas *et al.*[18] used specific primers for the detection of *Dicrocoelium dendriticum* single infections in snail tissues, and documented a higher accuracy of PCR over cercarial release. Later, Martínez-Ibeas *et al.*[54] designed a mtDNA multiplex PCR for identification and discrimination of *Caliophoron daubneyi* and *Fasciola hepatica* in the snail *Galba truncatula*, but they did not find natural double infections, neither by microscopy nor by PCR. As far as we know, our is the first study where species-specific primers have been designed in a duplex PCR for the accurate assessment of single and double infections with a blind sample of snail tissue. Results of our duplex PCR method have been statistically compared with those of the classical detection method, and shown to consistently outperform the latter. With this methodology, low parasite burden, prepatent, immature or covert infections, and death of the molluscs after collection do not prevent estimation of ‘true’ prevalence.

Curtis [11] noted that the magnitude of the influence of parasitism on snails used in experiments has often been ignored or underestimated. The consequences of underestimating the effects of parasitism are compounded if the prevalence of infection or the types of species combinations in multiple infections are underestimated [17], for example in the detection of potential seasonal changes [9]. Thus, determining true infection prevalences is extremely important. In the present study we demonstrate that up to 66.9% of the single and up to 80% of the double infections are overlooked when the commonly used classical emission method is employed to determine parasite prevalences. We demonstrate that molecular detection of single and double infections by single round duplex PCR strongly outcompetes the classical method; it also avoids misidentifications in case of morphologically similar species infecting the same host or when immature larval stages are present. Caron *et al.*[14] pointed out that prevalence appears higher with PCR-based methods than microscopy-based techniques, because the former is more sensitive. Cucher *et al*. [19] proved that the detection rate of PCR methods is statistically higher than that of shedding and dissection, but until now this difference had not been tested in a mixed infection parasite-snail system.

Two different ITS rDNA regions that can be used to identify single species unequivocally [55,56] were chosen, ITS1 for the Ebro species, and ITS2 for the Otago species, selecting highly variable and thus species-specific sequence regions (see Table 1). Some authors have proven that ITS2 is too conserved for distinguishing closely related taxa [39,57], with ITS1 showing greater divergences between species [58]. The greater sequence variation found in the ITS1 [39,59] also permits the detection of intra-specific patterns of variation [47] and the study of closely related species, while ITS2 is more appropriate for the analysis of more distant relationships [59] and can be used as marker at species or genus level [57,60,61]. Since both Ebro species belong to the same family, are morphologically very similar, and have highly similar ITS2 regions, primers were designed in the ITS1 regions. The Otago species can be easily differentiated morphologically as they belong to different families, thus primers were designed to amplify ITS2 regions.

The percentage of PCR positive samples not detected by emission, especially high for double infections, demonstrates the greater accuracy of the duplex PCR over the classical method. Cercarial emission misses many infections and underestimates true prevalence. The difference between the detection methods was higher for the Ebro samples, also showing much variability between years (17.9% in 2011 *versus* 60.1% in 2013) that could be due to immature infections of *C. labracis* during 2013. The percentage difference between methods for the Otago samples (9.9% in LP *versus* 20.6% in OB) highlights the differences of infection prevalences between sampling sites, as reported previously [29].

A noteworthy finding is the higher detection of double infections with the PCR method, implying that the prevalence of single infections is in fact lower. This is of special importance for research on parasite community structure. As an example, in the present study, simple infections that were found to be double infections represented 6.2% of the Ebro samples and 2.8% of the Otago samples. Detection failure is most likely due to immature infections, and the higher incidence of misidentified double infections in the Ebro samples must arise from problems with morphological identification of closely-related trematode species.

Thus, the few unusual findings can be explained by misidentification of the species, especially in the two opecoelid species infecting *G. adansonii* in the Ebro, since two morphologically similar larval stages infecting the same host may mask multiple infections [24]. After the removal of the snail tissue, a few “non-infected snails” in the Otago samples were found to have *Philophthalmus* sp. rediae; however, detection success by dissection is still much lower than that of the duplex PCR, especially regarding mixed infections not found by dissection. In any case, these results are included in the contingency table and the high power of detection of the PCR method has been statistically proven.

The difference between the methods in the case of *Philophthalmus* sp. (Otago samples) and for double infections in all samples is small at first sight, but their actual frequencies are also low, so that the higher detection levels when using the duplex PCR method is in fact unambiguous. Samples showing high agreement between both detection methods are probably the ones involving mature infections, which are easier to detect by the classical method. Ebro samples show generally a lower agreement between methods, due to the similarity in cercarial morphology between species making them harder to distinguish with the classical method. In such cases, the duplex PCR method is the best method to identify and detect infections. On the contrary, the Otago samples show generally a high agreement between methods, which confirms that the parasites are easier to differentiate. However, the agreement is far from perfect, which demonstrates that, even with morphologically dissimilar species, duplex PCR clearly outcompetes the classical method, allowing detection of immature infections.

Research on interactions between trematode species within snails generally focuses on immunity, altered attractiveness or interspecific antagonism. Kuris and Lafferty [2], in an exhaustive meta-analysis on interspecific interactions affecting the community structure of larval trematodes in snail hosts, concluded that as a general rule, fewer multispecies infections are observed than expected by chance (f_e_ > f_o_). We have applied the formula proposed by Cort *et al*. [6] and used in Kuris and Lafferty [2] for calculating the expected frequency of multiple infections (f_e_), and compared these with the observed prevalences (f_o_) obtained separately with the different detection methods. Generally, the data obtained by the classical detection method followed the rule f_e_ > f_o_, i.e. frequencies of double infections are lower than expected, suggesting competitive exclusion between trematode species in both the Ebro and Otago systems [2,4]. But when we calculated expected frequencies based on the duplex PCR method, a more complicated pattern emerged. With the exception of double infections in Oyster Bay, Otago, double infections were more frequent than expected by mere chance (f_e_ < f_o_). This involved parasite species having only sporocyst larval stages (Ebro), and a species with rediae interacting with another developing only sporocysts stage (Otago). Sousa [1,7] and Kuris [26] documented cases of positive or neutral associations between certain species, involving two sporocysts-only species or species with different larval stages (rediae *versus* sporocysts). Within-host interactions between trematodes in *G. adansonii* in the Ebro system have never been studied, while interspecific competition between *Philophthalmus* sp. and *M. novaezealandensis* is known to occur in the Otago system [25,27]. Some studies have shown that a rediae-sporocysts confrontation results in a patent decrease in the cercarial production of the subordinate species [23] causing double infections to be less frequent than expected. Perhaps competition between the two species in the Otago system remains relatively weak and does not really affect the probability of mixed infection. This hypothesis is in accord with our findings based on the duplex PCR method: if double infections are more frequent than expected by chance and competition is weak, this interspecific interaction may have little effect at the infracommunity level. The higher accuracy of the duplex PCR method can therefore change our interpretation of community structure of larval trematodes in snail hosts, by providing a clearer assessment of the importance of interspecific competition within the host and suggesting that other mechanisms may facilitate double infections.

Two notes of caution apply to these comments about double infections, however. First, the rough calculation of expected frequencies (f_e_) performed here provides only an approximation of species co-occurrences in these systems. A more exhaustive study with higher numbers of snails would be necessary to test the significance of differences between f_e_ and f_o_ in relation to interactions between species in mixed infections. Second, the duplex PCR method may also somewhat overestimate the frequency of double infections, by detecting the earliest stages of infection by one species, soon to be eliminated by the previously established competing species. In this sense, the two species will sometimes coexist very briefly in the same host, because infection by the subordinate species will not always become fully realised. Findings of double infections made possible by the greater sensitivity of the duplex PCR must therefore be interpreted with caution.

The inaccuracy of cercarial emergence as a sure sign of infection derives from infections that do not release mature cercariae at the time of study [17]. Because of temporal variability in cercarial production and species-specific emergence conditions [32,33,37,38], prevalences can only be obtained after long-term monitoring of cercarial shedding. Despite being time-consuming, the advantage of the classical shedding method is that it requires only basic laboratory equipment, like a microscope. The duplex PCR, on the other hand, is more costly as it requires DNA extraction and PCR reagents. However, several samples can be analyzed simultaneously in 96-well plates. Caron *et al.*[14] showed that an optimized PCR protocol allows the simultaneous analysis of up to 200 samples in less than 10 h, with a cost of about 0.3 Euro per snail. The obvious advantage is that the incubation period is skipped and samples can be analyzed immediately with low human error. In addition, snail tissue can be stored in 100% ethanol or as extracted DNA in the freezer until further analysis or for the possibility of future studies. Given its precision, the number of snail samples needed for accurate estimates of prevalence is also lower with the duplex PCR. The principal advantage of the duplex PCR remains its high sensitivity and specificity because the sequence amplified is always accurately targeted with primers [14], so that immature and covert infections can be also identified.

## Conclusions

Our species-specific ITS-based PCR assay for the calculation of ‘true’ prevalences may be extrapolated to other systems, even those including more than two parasite species. We feel this method should be considered as an additional tool for determining prevalences of larval stages with high accuracy. The findings presented here also challenge previous conclusions based on cercarial emission studies, concerning interspecific competition in mixed infections, parasite population parameters (prevalence and intensity of infections in snails) and seasonal fluctuations in parasite recruitment into snail populations.

## Competing interests

The authors declare that they have no competing interests.

## Authors’ contributions

AB-T and ASH designed the study. AB-T carried out the field activities, analyzed the data and prepared the manuscript. ASH supervised the molecular study and RP the statistics. ASH, RP and JAR critically revised the manuscript for intellectual content. All authors read and approved the final manuscript.
